# Poor follow-up rates at a self-pay northern Indian tertiary AIDS clinic

**DOI:** 10.1186/1475-9276-6-14

**Published:** 2007-10-24

**Authors:** Duncan Smith-Rohrberg Maru, Deepika C Khakha, Mohammad Tahir, Sanjay Basu, Surendra K Sharma

**Affiliations:** 1 AIDS Program, Department of Internal Medicine, All India Institute of Medical Sciences, New Delhi, 11029, India; 2 Department of Medicine, Yale University School of Medicine, New Haven, CT, 06510, USA

## Abstract

**Background:**

In many developing countries, out-of-pocket payment remains a primary mechanism by which patients infected with HIV access treatment. In India, this has been changing as the National AIDS Control Organization (NACO) has been rolling out free antiretroviral therapy throughout the country since 2004. The vast majority of patients, however, remain without access to free medicines.

**Methods:**

A retrospective chart review was performed on data obtained from a registry of ninety-three (93) patients attending a self-pay clinic at the All India Institute of Medical Sciences in Delhi, India. Multivariable Cox proportional hazard and logistic regression models were explored to assess the relationship between lost-to-follow-up status and the predictor variables: age, sex, household income, baseline CD4 count, and distance from clinic.

**Results:**

Lost-to-follow-up rates were very high; 68% (63/93) were lost-to-follow-up till the time of chart review, including 59% (55/93) who were lost within one year. In both regression models, younger age, low baseline CD4 counts, and low income level were significantly associated with increased risk of lost-to-follow-up. Additionally, there was a significant interaction between income and CD4 counts. The patients with both low CD4 counts and low income level were more likely to be lost-to-follow-up than would be predicted by each covariable alone.

**Conclusion:**

In this small cohort of AIDS patients attending a self-pay antiretroviral clinic at a large tertiary care center in Delhi, India, follow-up rates were quite poor. Poorer patients tended to present to clinic with more depressed CD4 counts and were less likely to be retained in care. These findings indicate that greater strides must be taken to improve the recruitment and retention of poor patients. The expansion of free antiretrovirals is one step among many necessary to achieve this objective.

## Introduction

Since April 2004, the National AIDS Control Organization (NACO) has been funding medications and services to be delivered through free antiretroviral (ARV) clinics throughout India[1]. This has been a welcome change for the approximately five million Indians already living with HIV/AIDS[2], many of whom now have a possibility of accessing life-saving medications. Previously, only patients with financial resources to pay, had access to ARV services mostly through India's largely unregulated private sector, where more than 75% of all healthcare expenditures take place[3]. Patients additionally could receive ARV services through a limited number of tertiary care government hospitals where consultation was available at subsidized prices but where medication costs were to be born by patients; other patients did not receive services. For many patients in rural areas or in states where free ARVs have been slow to come, this still remains the reality. Additionally, many laboratory services and other medications necessary for comprehensive AIDS care are not available for free.

NACO originally focused their resources on the six high-prevalence states of Andhra Pradesh, Karnataka, Maharashtra, Tamil Nadu, Manipur, and Nagaland, as well as the national capital territory and seat of NACO, Delhi. Since then, ARV roll-out has been expanding to other lower-prevalence states with more concentrated epidemics. Owing to logistical concerns over distributing ARVs and providing care in more rural areas, much of the ARV roll-out has focused on urban centers associated with large government hospitals. While this has advantages, for example, in greater ease of access to laboratory services, this has made access to care challenging, if not impossible, for some patients, especially among rural or sub-urban poor patients. Even in the era of free ARVs in India, many patients must use significant financial resources to pay for their care.

Very few studies to date have assessed follow-up rates among patients living with HIV/AIDS in India. One ethnographic study among patients in Chennai, Tamil Nadu (southern India) highlighted the extreme measures that patients often take – selling personal and family property, for example – to cover the costs of medicines. Among the sixty patients investigated, thirty-two percent reported having taken a self-imposed drug holiday owing to financial difficulties[4]. Another study by the same group of researchers, but among a different cohort of three-hundred patients from Chennai, reported high levels of adherence, although cost was the most common cause of non-adherence (32%)[5].

To examine the challenges of fee-for-service AIDS care in the northern Indian context, we undertook a retrospective chart review of a small cohort of patients attending a self-pay outpatient clinic at the All-India Institute of Medical Sciences (AIIMS), a tertiary care center in Delhi. This was done during the transition period from self-pay to free ARVs, following the designation of AIIMS as a new NACO free ARV center. This analysis was thus undertaken as a way to assess the challenges of the self-pay system, both to examine how the new clinic structure might address these issues and to inform the public health issues in the many areas where free ARVs have yet to arrive.

The variables that were available for analysis included: age, sex, distance from clinic, household income, and CD4 count at ARV initiation. We hypothesized that lower CD4 count as an indicator of more advanced disease, lower household income as an indicator of poverty level, and distance from clinic as an indicator of a barrier to access would be associated with increased risk of the patients' lost-to-follow-up.

## Methods

We retrospectively reviewed the charts of all HIV-infected patients (n = 93) treated with antiretrovirals at the AIIMS clinic during the years 2001 to 2004. AIIMS is a large tertiary care government hospital in India's capital city, and caters to a wide variety of patients from all socioeconomic backgrounds. Although medical care and consultations are typically provided for free or at low cost, most medications are obtained by patients at market prices. This was the case during the duration of the study period; the study period ceased following the introduction of the new NACO-sponsored free ARV clinic. Patients were initiated on ARVs if their CD4 counts were less than 200 cells/microliters, if they had AIDS-defining illnesses, or at the discretion of the senior physician.

At intake, clinic staff administered a questionnaire to probe their demographic background, prior medical treatment, and HIV risk. This included monthly household income ("household income") and time in hours it took to reach the clinic by available mode of transportation ("distance from clinic"). The nurse practitioner also took responsibility for consenting and counseling the patients, and monitoring their medications.

Patients were referred from various disciplines within AIIMS or health care providers outside AIIMS, even from other states. Regimens were typically nevirapine and lamivudine, with either stavudine (80%) or zidovudine (8%); the remaining patients were on efavirenz-based regimens, with lamivudine and either zidovudine or stavudine; one patient received nevirapine with indinavir and zidovudine. All patients received baseline CD4 measurements. Owing to cost, CD4 counts were not consistently available at follow-up. Lost-to-follow-up was defined by missing at least two monthly appointments and never returning to clinic. As patients were typically not followed by phone or mail, the clinical team was unaware of what happened to such patients.

Data analysis was conducted with SAS version 9.1 (Carey Institute, North Carolina, USA). All statistical tests were assessed at an alpha = 0.05 level. All patients started on ARVs had baseline CD4 counts that could be used as independent predictor variables. Owing to the lack of CD4 counts or HIV viral loads available at follow-up, neither of these could serve as outcomes variables. As such, survival analysis was performed with time-to-failure calculated from the time when patients started HAART to the time when they were lost to follow-up or were censored (owing to the start of the new free clinic), rounded to the nearest month. A Cox proportional hazards multiple regression model was attempted to fit the data using the demographic variables as well as baseline CD4 count. There were five patients for whom income data was not available; for these patients, the overall mean income level was given (i.e., their centered income was set to 0). The proportionality assumption was tested by assessing for significance of the time-dependent covariables (using the natural logarithm of time). During the model building process, the log-likelihood ratio (deviance) was used to compare two models and decide whether a variable should be kept or dropped.

To check the findings of this analysis, a multivariable logistic model was attempted to fit the same data, comparing patients who were lost to follow-up versus those who were retained in care through the opening of the new ARV clinic. Preliminary bivariable analyses between outcome and predictors were undertaken using two-tailed Fisher's exact test for categorical variables and independent samples t-test for continuous variables. Pearson's correlation coefficients were used to make pair-wise comparisons between predictor variables.

All odds ratios and hazards ratios were adjusted to the units of the variable in question for ease in interpretation (per 25 cells/microliter for CD4, per 500 Rs for income, and per 5 hours for distance from clinic). Note that this does not alter significance testing, only interpretability of the resulting odds ratios and hazard ratios.

## Results

**Figure 1:**
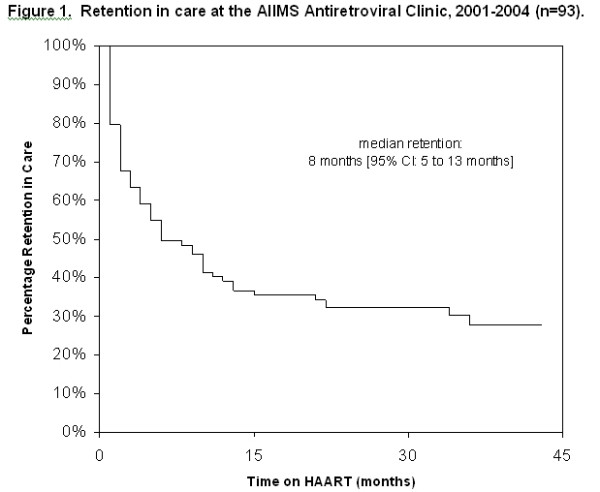
Survival function time-to-LTFU.

Basic demographic characteristics are shown in table 1. For those patients for whom counts were available at 6 months follow-up (n = 25), the median CD4 increase was 159 cells/microliter (Interquartile Range 34 to 254). Owing to the small number of patients with follow-up laboratory data, no further analyses were undertaken on follow-up CD4 counts. Following ARV initiation, 63 of 93 (68%) missed at least two appointments and never returned. A total of 55 patients were lost within 12 months or less, including 11 patients who did not return after their first post-ARV-initiation visit. Patients came not only from Delhi (n = 31) but also from several neighboring states, most prominently Utter Pradesh (n = 21), Haryana (n = 16), Bihar (n = 15), states with varying levels of low health infrastructure. Women tended to live closer to the clinic than men, though not significantly so (4.32 versus 7.16 hours, p-value = 0.11). Age, income, distance from clinic, and CD4 count were all significantly associated with lost-to-follow-up in preliminary bivariable analyses (table 2). The survival curve for the whole sample, assessing time to lost-to-follow-up (LTFU), shows a rapid decline within the first year following ARV initiation (Figure 1). Income, distance from clinic, and CD4 count at baseline were highly correlated; in multivariable analyses, we thus corrected for this by centering these variable around the mean.

**Table 1:** Characteristics of patients receiving ARVs at AIIMS self-pay clinic (n = 93)

Characteristic	Value
Age in years, Mean +/- SD	34.4 +/- 8.0
Sex, females, n (%)	19 (20.4%)
Distance from clinic in hours, Mean +/- SD	6.5 +/- 7.9
Monthly Household Income in Rs and $, Median (IQR)	Rs.3000 (2500 to 5000) US$68 (56.8 to 113.6)
Median CD4 Count at ARV initiation in cells/μL (IQR)	83 (57 to 146)
Median Time of Follow-Up in Months (IQR)	8 (3 to 22)

**Table 2:** Bivariable analysis of predictor variables

Characteristic	Continued Care (N = 30)	Lost to Follow Up (N = 63)	P-Value
Age in years, Mean +/- SD	37.2 +/- 9.2	33.0 +/- 7.1	0.04
Sex, females, n (%)	7 (23.3%)	12 (19%)	0.78
Distance from clinic in hours, Mean +/- SD	4.2 +/- 5	7.7 +/- 8.8	0.02
Monthly Household Income in Rs and $, Median (IQR)	Rs.5000 (3000 to 5500) US$114 (68 to 125)	Rs.2500 (2000 to 3500) US$57 (45 to 80)	0.0006
Median CD4 Count at ARV initiation in cells/μL (IQR)	129.5 (67.0 to 179.0)	72 (41 to 129)	0.005
Median Time of Follow-Up in Months (IQR)	33.9+/-15.5	7+/-8.4	<0.0001

For the Cox mulitivariable regression, the proportional hazards assumption was violated only for the variable of CD4 counts at baseline (Deviance = 4.9, p-value = 0.03). As such, this variable was retained in the model, whereas the other demographic variables were dropped, since there was neither a plausible explanation for time-dependence nor did any of their parameter estimates approach significance. To further explore the relationship between the main effects, second-order interaction terms were thus added in the model. The only interaction term found to be significant was that between CD4 count at baseline and income (LR difference = 9.8, p-value = 0.002). The final result of this model building process is shown in Table 3.

**Table 3:** Hazard ratios for cox regression model

**Main Effects Model**		
Variable	HR (95% Confidence Interval)	p-value
Age	0.96 (0.93 to 1)	0.02
Sex	0.82 (0.42 to 1.6)	0.71
Monthly income	0.89 (0.82 to 0.97)	0.004
Distance from clinic	1.00 (0.96 to 1.16)	0.92
Baseline CD4 Count	0.91 (0.8 to 1.03)	0.11
**Model Including Interaction Terms**		

Variable	HR (95% Confidence Interval)	p-value
Age	0.96 (0.93 to 0.99)	0.02
Sex	0.90 (0.46 to 1.76)	0.71
Monthly income	0.86 (0.79 to 0.94)	0.004
Distance from clinic	0.99 (0.95 to 1.02)	0.92
Baseline CD4 Count	1.49 (1.11 to 2.01)	0.11
CD4 * Time Interaction	0.91 (0.81 to 1.04)	0.03
CD4 * Income Interaction	0.94 (0.9 to 0.99)	0.01

Note: Hazard Ratios represent the increased relative risk associated with a unit increase in the covariate; for age, 1 year; for monthly income, 500 Rs; for distance from clinic, 5 hours; for CD4 counts, 25 cells/microliter.

To confirm these results, a multivariable logistic regression model was developed; as before, the log likelihood ratio was used to compare models in a forward stepwise fashion. In the multivariable regression case, the interaction term between CD4 count at baseline and income did not reach significance (Deviance = 1.8, p-value = 0.16). However, this term was kept in the model for comparability. The final results are shown in Table 4. Again, income and age were significant predictors of lost-to-follow-up status, as was the CD4-by-income interaction.

**Table 4:** Odds ratios for logistic regression model

**Main Effects Model**		
Variable	OR (95% Confidence Interval)	p-value
Age	0.94 (0.88 to 1)	0.02
Sex	0.87 (0.26 to 2.96)	0.71
Monthly income	0.82 (0.71 to 0.95)	0.004
Distance from clinic	1.06 (0.72 to 1.56)	0.92
Baseline CD4 Count	0.79 (0.64 to 0.98)	0.11
**Model Including Interaction Terms**		
Variable	OR (95% Confidence Interval)	p-value
Age	0.92 (0.86 to 0.99)	0.02
Sex	0.77 (0.21 to 2.9)	0.71
Monthly income	0.76 (0.63 to 0.92)	0.004
Distance from clinic	0.98 (0.66 to 1.46)	0.92
Baseline CD4 Count	1.80 (0.87 to 3.7)	0.11
CD4 * Income Interaction	0.89 (0.81 to 0.99)	0.03

Note: Hazard Ratios represent the increased relative risk associated with a unit increase in the covariate; for age, 1 year; for monthly income, 500 Rs; for distance from clinic, 5 hours; for CD4 counts, 25 cells/microliter.

## Discussion

In this small cohort of geographically-diverse patients receiving self-pay HIV care at a tertiary care center in northern India, high lost-to-follow-up rates were seen. In multivariable Cox and logistic regression models, we found that lost-to-follow-up patients were younger in age, had lower CD4 counts at presentation, and had lower monthly incomes. An interaction term between income and baseline CD4 count was significant in both models. This suggests that poorer patients initiated ARV therapy at a more progressed disease state, and when they did enter care, they tended not to be retained in care. The interaction term suggests that poorer patients with lower CD4 counts had worse follow-up rates than would be predicted by their CD4 counts and income levels alone. That is, the model of healthcare delivery that was being undertaken at that time was least effective for the most sick and poor patients.

Although women constitute approximately half of the Indian epidemic, the clinic only recruited 20% women. While this study cannot directly address this issue, it is likely due to their limited mobility compared to men. Indeed, those women who did come into care tended to live farther away from the clinic, though not significantly so. Further studies should address this issue, although it is fairly intuitive that local provision of care should improve both recruitment and outcomes of HIV-positive women.

The most prominent limitations of this study are its retrospective nature, its small sample size, and the high variances inherent in collecting social data like income and distance from clinic. Few generalizations should be made from these preliminary results. Additionally, per capita income of the patient would have been a more useful indicator of economic status than household income. Unfortunately, we did not collect the data in a way that could assess this; as such, the association of economic status and poor follow-up must be considered tentative.

On the whole, however, our results do suggest that ARV delivery methods in India must be improved to ensure good patient outcomes and ward off drug resistance. Excellent results have been demonstrated in resource-poor settings, including India, with decentralized, locally-provided care with strong social support and community healthworkers [5-8]. Even in this cohort, the improvements in CD4 counts for the small number of patients for whom these were available at 6 month follow-up were consistent with other studies[9]. Private providers will continue to play an important role in the Indian health system[10], and engaging such providers to deliver high-quality care to poor patients is central to improving equity[11,12]. Financing mechanisms to decrease financial burden on patients are feasible in developing countries[13]. Such mechanisms must serve to better recruit and retain poorer patients into AIDS care and treatment.

## Competing interests

The author(s) declare that they have no competing interests.

## Authors' contributions

DSR performed data analysis and drafted the manuscript.

DCK collected the data, edited the manuscript, and provided nursing care to patients.

MT assisted with data analysis and edited the manuscript.

SB assisted with data analysis and edited the manuscript.

SKS conceived the study, manages the clinic, and edited the manuscript.
